# Pregnancies following the use of sequential treatment of metformin and incremental doses of letrozole in clomiphen-resistant women with polycystic ovary syndrome

**Published:** 2012-01

**Authors:** Azam Azargoon, Jafar Alavy Toussy, Fakhry Fakhr Darbanan

**Affiliations:** 1 Department of Gynecology and Obstetrics, Semnan University of Medical Sciences, Semnan, Iran.; 2 Department of Pathology, Semnan University of Medical Sciences, Semnan, Iran.

**Keywords:** *CC-resistant*, *Induction ovulation*, *Letrozole*, *Metformin*, *PCOS*

## Abstract

**Background**: Clomiphen citrate (CC) is the first line therapy for women with infertility and poly cystic ovary syndrome( PCOS). However, 20-25% of women are resistant to CC and do not ovulate.

**Objective:** The objective of this study was to evaluate the efficacy of sequential treatment of metformin and incremental doses of letrozole in induction of ovulation in cases of CC-resistant PCOS patients.

**Materials and Methods**: In this prospective before-after study, we enrolled 106 anovulatory PCOS women who failed to ovulate with CC alone from Amir-Almomenin University Hospital in Semnan, Iran. After an initial 6-8 weeks of metformin treatment, they received 2.5 mg letrozole daily on days 3-7 after menes. If they did not ovulate with 2.5 mg letrozole, the doses were increased to 5 to 7.5 mg daily in subsequent cycles. The main outcomes were ovulatory rate, pregnancy rate and cumulative pregnancy rate.

**Results**: 13.33% of patients conceived with metformin alone. Ovulation occurred in 83 out of remaining 91 patients (91.2%). 78.02% of patients responded to lower doses of letrozole. Cumulative pregnancy rate was 60/ 105 (57.14%).

**Conclusion**: We suggest that treatment in CC-resistant PCOS patients should begin at first with lower doses of letrozole and could increase to the higher dose depending on the patient response before considering more aggressive therapeutic alternatives such as gonadotropins.

## Introduction

Polycystic ovary syndrome (PCOS) is one of the most common causes of anovulatory infertility and affects 5- 10% of women of reproductive age. It is frequently associated with insulin resistance and compensatory hyperinsulinemia.Women with this syndrome of chronic anovulation and hyper androgenis are at increased risk of obesity, diabetes, infertility, and miscarriage. Moreover, there is no clear evidence as to which treatment intervention is more effective for restoring fertility (1, 2). 

Clomiphen citrate (CC) is the first line therapeutic modality for women with infertility and PCOS. CC is accumulated in the body with low clearance rate and long half- life (5 days). Significant plasma concentrations of the active Zu isomer of CC can be detected up to 6 weeks after administration (2, 4). However, 20-25% of PCOS women fail to ovulate with incremental doses of CC and are resistant to CC. 

In addition, clinical data revealed a discrepancy between ovulation rates (75-80%) and conception rates (30-40%) during CC treatment (3, 4). Ovulation induction with gonadotropins is the standard treatment for clomiphene- resistant (CR) women; however, this is expensive and has added risks of ovarian hyper stimulation and multiple pregnancies (2, 5). 

Therefore a simple oral treatment that could be used without risk of hyper stimulation and with minimal monitoring would be the preferred therapy. Letrozole is a newly designed selective aromatase inhibitor, with the above characteristics, and can be used to induce ovulation in infertile women with polycystic ovary syndrome (4, 6). Several studies have also been performed concerning the beneficial role of letrozole in CC- R patients, but most of them used only 2.5 mg letrozole for treatment (3, 4, 6, 7). 

Hyper insulinemia is one of the diagnostic features of PCOS and patients with PCOS are found to have resistance to either endogenous or exogenous insulin (8). It is also known that patients with PCOS and insulin resistance are often resistant to ovulation induction and the suggested treatment includes the use of insulin sensitizers such as biguanides (metformin) and thiazolidindions (5, 9). 

The beneficial effects of metformin have been shown in PCOS patients by improving pregnancy rate and the metabolic situation and by decreasing the complications of pregnancy such as gestational diabetes (10). In addition, the beneficial effects of combined metformin-clomiphene therapy have also been reported in CC-R patients (2, 5, 6, 8, 10). However, no studies have yet evaluated the effects of sequential therapy of metformin with incremental doses of letrozole in CC-R patients. Considering of beneficial effect of metformin and letrozole in CC-R women with PCOS, we decided to perform this large study with these patients, to determine with which doses of letrozole the maximum response would occur.

## Materials and methods

One hundred and eleven women were enrolled in this before-after study from infertility clinic at Amir-Al-Momenin Hospital, Semnan, Iran from June 2005 to December 2008. All patients were informed about the study and possible complications of the drugs by a specialist and signed consents were received from them. This study was supervised and approved by the Research Council and Ethical Committee of Semnan University of Medical Sciences.

The inclusion criteria included women who were 18-24 years of age, with period of infertility more than 1.5 years and normal serum prolactin and thyroid function tests, they had documented patent tubes by hystero salpingography, had no other infertility factor, and failed to ovulate with a dose of CC of 150 mg/day for 5 days from day 3 of the period. Women were excluded from the study if they were diabetic, were taking any medication that could influence carbohydrate metabolism, had hypertension or abnormal renal or liver function tests, and had undergone ovarian drilling. Hirsutism was diagnosed when the Ferriman and Gallway score was>8 (11). 

The diagnosis of PCOS was according to the Rotterdam Criteria (12). A trans- vaginal ultrasound examination with a vaginal transducer 6.5 MHZ (Honda, Japan) was performed to exclude any pelvic pathology before treatment with metformin.

**Study design**

Metformin was started after two months of CC to allow for the washout of the latter. All women were examined clinically and their weight, height and body mass index (BMI) recorded. All patients received 1500 mg metformin (Glucophage, Merck, West Drayton, UK) daily for 6-8 weeks. 

The dose of metformin was built up gradually over a 3 weeks period, beginning at 500 mg daily with main meal for 1 week, followed by 500 mg twice daily for 1 week and increased to 500 mg three times daily to minimize side effects. If pregnancy occurred, metformin was continued for another 8 weeks. In case of failure of pregnancy after the end of this period, metformin was continued and patients were given 2.5 mg letrozole (Femara, Novartis, Quebec, Canada) for 5 days starting from day 3 of their menstrual cycle. 

Ovarian follicular response was monitored by transvaginal sonography every other day from day 10 of the cycle by a single sonographist. When at least one follicle reached ≥18 mm in diameter, 10000 IU of HCG (Pregnyl; N.V. Organon, OSS, Netherlands) was given intramuscularly and timed intercourse was advised (every other day for one week starting after receiving HCG). Endometrial thickness and number of mature follicles were determined on the day of HCG administration. If there was no follicle ≥12mm by day 16, the cycle was presumed to be anovulatory and monitoring was discontinued. 

Clinical pregnancy was defined as the detection of at least one gestational sac on transvaginal ultrasound examination starting one week after the missed period. In cases of ovulation without pregnancy, the patients were advised to continue metformin and to participate in other two similar cycles of therapy with 2.5 mg letrozole. When ovulation did not occur with 2.5 mg letrozole, its dose was increased to 5 and then 7.5 mg and the same treatment protocol were used for each dose.

**Outcome measures**

The primary outcome measures were ovulation, pregnancy and cumulative pregnancy rates in different doses of letrozole. 

The mean number of follicles ≥18 mm, the mean of follicular size and endometrial thickness on the day of HCG administration were secondary outcome measures.

**Statistical analysis**

All the data entered to the SPSS software (Version 11.5.0, © SPSS Inc.), T-test (or Mann-Whitney test if needed) and ANOVA for quantitative variables and Chi-square test (and Fisher exact test if necessary) for qualitative variables used. 

p<0.05 considered significant in all tests. We used Epi Info statistical software (version 6.4, WHO and CDC) for calculation of some ratios and their confidence intervals too.

## Results

One hundred eleven women were eligible but 5 women refused to participate in the study, so 106 patients entered the study. Table I shows demographic, clinical and hormonal features of all women involved in this study. One patient developed generalized rash with metformin and was excluded from the study. 14 of 105 patients (13.33%) conceived with metformin alone. From 14 pregnancies, 2 cases (14.28%) were aborted and 2 cases (14.28%) were preterm. 21 of 106 patients (19.81%) had side effects with metformin including: nausea in 8 (7.5%), dizziness in 5 (4.7%), anorexia in 2 (1.8%), diarrhea in 2 (1.8%), dizziness and nausea in 2 (1.8%), vomiting in 1 (0.9%) and rash in 1 patient (0.9%); 8 of 106 patients had to decrease the dosage of metformin to 1000 mg or 500 mg daily due to side effects.

The remaining 91 patients who did not conceive with metformin continued metformin use and letrozole 2.5 mg was started for them. Ovulation occurred in 37 out of 91 patients (40.65%) and pregnancy rate per ovulatory cycle was 21/ 78 (26.92%) (Table II). From 21 pregnancies, 2 (9.25%) were aborted, 2 (9.25%) were preterm and 1 (4.7%) was a twin pregnancy. 

The remaining 54 patients who had not ovulated with 2.5 mg letrozole continued metformin use and 5 mg letrozole was started for them. In this group, ovulation occurred in 34 out of 54 (62.96%) patients and pregnancy rate per ovulatory cycle was 21/70 (30 %) (Table II). Of 21 pregnancies, 4 (18.5%) were miscarriages, 1 (4.7%) was preterm and 3 (14.1%) were twin pregnancies. 

Except one patient, the remaining 19 patients who had not ovulated with 5 mg letrozole entered into the last stage of this study (metformin+7.5 mg letrozole). Ovulation occurred in 12 out of 19 (63.15%) patients. Pregnancy rate per ovulatory cycle was 4/32 (12.5%) (Table II). The mean number of follicles ≥18mm, the mean follicular size and endometrial thickness on the day of HCG administration of different doses of letrozole are shown in Table II. 

**Figure 1:**
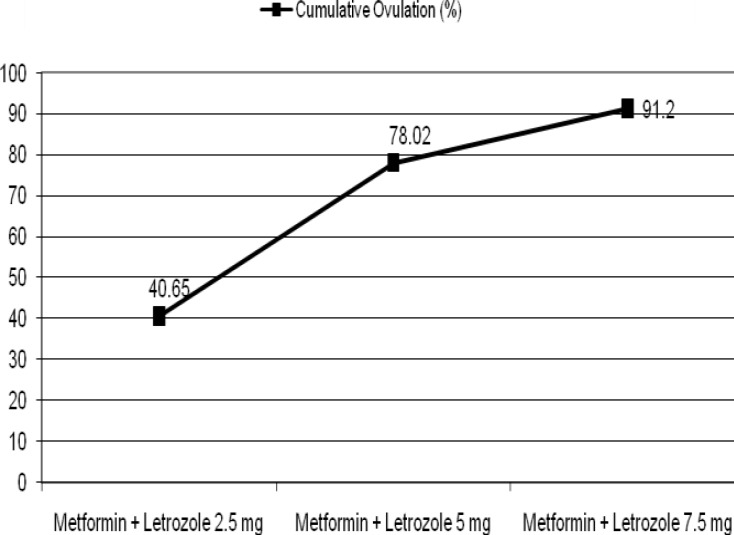
Cumulative ovulation rate in women who treated with letrozole and metformin (n=91).

**Figure 2:**
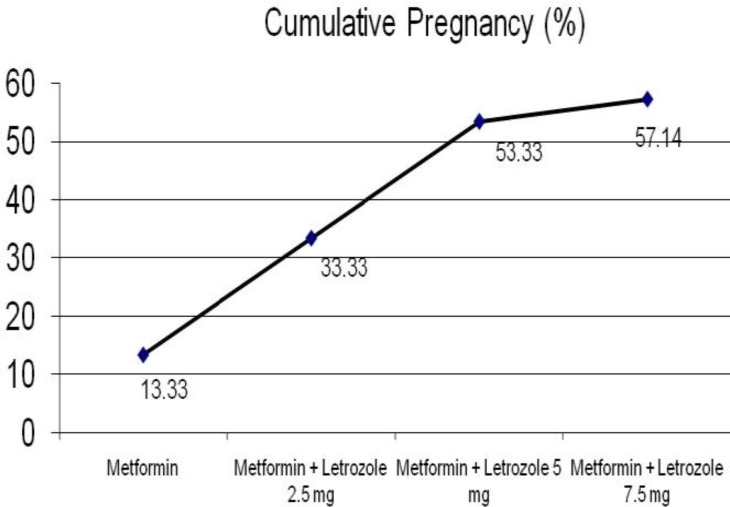
Cumulative pregnancy rate in women with CC-resistant PCOS who treated with letrozole and metformin (n=105).

In the end of this study ovulation rate with letrozole was 83/91 (91.2%). 71 of 91 patients (78.02%) responded to doses of 2.5mg and 5mg of letrozole. Only 20 (21.97%) of patients needed the higher doses of 7.5mg. In the end, 8 out of 91 patients (8.79%) remained anovulatory (Figure 1). In the end of this study, the cumulative pregnancy rate was 60 out of 105 patients (57.14%). Of 60 pregnancies, 10 (16.7%) were aborted, 5 (8.3%) were preterm, and 45 (74.9%) were full term. Cumulative pregnancy rate during treatment are depicted in figure 2. 

**Table I:** Main demographic, clinical and hormonal characteristics of the patients

**Variables **	**All patients**
Age (years	25.65 ± 4.79*
BMI (kg/m^2^)	26.96 ± 4*
Duration of infertility (years)	3.47 ± 2.92*
Primary infertility N (%)	78 (73.6)
PCO feature in sonography in both ovaries N (%)	84 (79.2)
**Menstrual pattern:**	
Oligomenorrhea N (%)	66 (62.26%)
Amenorrhea N (%)	40 (37.73%)
Hirsutism N (%)	39 (36.79%)
LH	8.30 ± 6.26*
FSH	5.65 ± 2.88*
LH/FSH	1.79 ±1.84*

* Note: are mean±SD.

**Table II:** Characteristics of treatment cycles in the different doses of letrozole

**Variables**	**Letrozole 2.5 mg**	**Letrozole 5 mg**	**Letrozol 7.5 mg**
On the day of HCG			
No of follicles ≥ 18 mm	1.54 ± 0.73*	1.47 ± 0.7*	1.46 ± 0.68*
Size of follicles (mm)	19.14 ± 2.9*	20.1 ± 3.03*	19.5 ± 3.06*
Endometrial thickness (mm)	7.44 ± 1.72*	7.76 ± 1.84*	6.64 ± 3.48*
Ovulation N (%)	37/91 (40.65%)	34/54 (62.96%)	12/19 (63.15%)
Pregnancy/cycle N (%)	21/78 (26.92%)	21/70 (30%)	4/32 (12.5%)

* Note: are mean ± SD.

## Discussion

Clomiphen citrate is the treatment of choice for the anovulatory infertile women with PCOS, though about 20% of these women do not respond. Ovulation induction with gonadotropins is the standard treatment for CR women. However, this approach is associated with complications and has the added disadvantage of high cost and need for an alternative, less expensive therapy (2, 5). Metformin has been shown to have beneficial effects on ovarian function and hormonal milieu. In 1994, Velazquez *et al* published the first report on the use of metformin in women with PCOS. 

In this case series, 26 women were given metformin for 8 weeks and showed improved insulin sensitivity, lowered serum testosterone concentration (by 50%) and increased SHBG. Furthermore, three pregnancies occurred (13). Subsequently, several studies were published on the beneficial effects of metformin in women with PCOS (1, 2, 5). 

With respect to metformin and CC in women with CC-R PCOS, Nestler *et al* showed that 19 out of 21 (90%) obese women with CC-R PCOS ovulated in response to metformin and CC therapy compared with 2 out of 25 (8%) women in corresponding placebo and CC control group (14). 

Several studies also demonstrated significantly higher ovulation rates in women treated with metformin and CC as compared with the placebo and CC group in CC-resistant women (2, 10, 15). In contrast, Sturrock *et al* did not report benefits from metformin therapy in CC-R PCOS women (16). Subsequently, in a meta-analysis it was shown that the addition of metformin in the CC resistant patients is highly effective in achieving ovulation induction (17). In the present study, metformin pre-treatment (6-8 weeks) followed by letrozole in clomiphene- resistant PCOS patients. With metformin alone, pregnancy rate was 14/ 105 (13.33%) with an abortion rate of 14.28%. In Neueu’s study of PCOS women who were treated with metformin, pregnancy rate was 45.6% during the 1year of treatment and abortion rate was19.2 % (1). In Sohrabvand’s study, only one patient conceived with metformin alone (6).

Metformin can cause gastrointestinal side effects. The occurrence of side effects in this study was lower than that observed in Heard’s study (19.8% versus 31%); it may be due to the administration of metformin 500 mg of two times initially at Heard’s study (18).

In this study, one patient discontinued therapy as in Heard’s study (18). In Neveu’s study four (4%) patients discontinued metformin due to unacceptable gastrointestinal side effects and 17.3% of patients had to decrease the doses of metformin (1). In this study, 7.5% of patients (8/ 106) had to decrease their doses of metformin. But like several other studies, these side effects were mild and tolerated and presented in the first 2 weeks of therapy (1, 8, 18). 

Some of these patients conceived while receiving lower initial doses of metformin. This showed that ovulation with metformin was independent of the dosage. This result was similar to the results of Neveu *et al* and Heard *et al *(1, 18). In the case of pregnancy, metformin was continued for the first 8 weeks of pregnancy and we did not detect any congenital anomalies in neonates. In agreement with several studies in a meta- analysis, our data is reassuring that the use of metformin before and at least during the first 8 weeks of pregnancy does not compromise the health of either mother or fetus (19).

Letrozole is a simple, inexpensive and safe alternative to CC that can cover the goal of ovulation induction to induce mono-follicular development. Mitwally and Casper had reported the novel use of aromatase inhibitor letrozole for inducing ovulation in anovulatory women with PCOS and for augmenting ovulation in ovulatory infertile women (4). 

In this study, ovulation occurred in 37/ 91 patients (40.65%) who used 2.5 mg letrozole (Table II). Ovulation rate was similar to Elnashar’s study (3) but was lower than that obtained by other studies. Mitwally and Casper had ovulatory rates of 75 % (4), Alomari *et al* had an ovulatory rate of 87.5% (7) and Sohrabvand had a rate of 90.57% (6). These variable results can be explained by the fewer numbers of the patients, type of selection and definition of clomiphene resistance in other studies.

The present study pregnancy per induced ovulatory cycle was 26.92 % (Table II). This result was similar to the results of Mitwally and Casper (25%) (4), Alomari *et al* (27.27%) (7) and Elnasher *et al* (25%) (3). During 2.5 mg letrozole treatment the mean number of mature follicles ≥18 mm and the mean of follicular size were 1.54±0.73 and 19.14±2.9 mm, respectively (Table II). The limited number of mature follicles decreases the risk of multiple pregnancies and ovarian hyper stimulation syndrome. 

These results are in agreement with the results of other studies (3, 6, 7). Therefore, letrozole treatment does not need intensive monitoring compared to CC and gonadotropins. In this study, mean endometrial thickness on the day of HCG administration was 7.44±1.72 mm (Table II), which was similar to the results achieved by Mitwally *et al *(4), Alomari *et al* (7), and Sohrabvand *et al* (6). Thus the endometrium was of adequate thickness to allow implantation. 

Because serum clearance of letrozole is faster than CC and dose not leads to a decrease in the estrogen receptors, it is probable that letrozole does not produce deleterious effects similar to that found with CC on the endometrium. With 5mg letrozole, ovulation occurred in 34 (62.96%) of patients and pregnancy per ovulatory cycle was 30% (Table II). The study performed by Metawie *et al*, comparing 5 mg letrozole with CC in CC-R PCOS patients, showed that pregnancy occurred in 17.5% of cases (20). As seen pregnancy rate is higher in our study. This can be related to using a combination of metformin and letrozole in our study.

Thus; it seems that a combination of metformin and letrozole is better than letrozole alone as in Sohrabvand *et al* study (6). With 5 mg letrozole the mean number of follicles≥ 18 mm, and endometrial thickness were 1.47±0.7, and 7.76±1.84 mm, respectively (Table II). These results were in agreement with the results by Al Fadhli *et al* (1.3±0.1, 7.8±0.3 mm). 

Al Fadhli *et al* compared 5mg letrozole with 2.5 mg use in women undergoing superovulation and IUI cycles and concluded 5 mg letrozole was associated with higher follicles and pregnancy rates than 2.5 mg. They also suggested that if mono- ovulation is desirable in ovulation induction, in superovulation we should prefer to have a higher number of follicles, and the 5 mg daily dose appears to be proper dose (8). 

The mean number of follicles ≥18mm, and endometrial thickness in patients with 7.5 mg letrozole on the day of HCG administration were 1.46±0.68, and 6.64±3.48 mm, respectively (Table II). In Al-Fozen’s study, these were 1.3±0.29 and 7.1±0.2 mm in patients with superovulation and IUI and pregnancy per cycle was 11.5% (21). 

Alfozen *et al* and Al-Fadhli *et al* also concluded the ideal dose of letrozole for ovulation induction or superovulation is still unknown (8, 21). In this study one pregnancy in patients who used 2.5 mg letrozole and 3 pregnancies in those who used 5 mg were twins. 3 of twins were in patients with 2 mature follicles and one in patient with 3 follicles. This is not similar to other studies. Twin pregnancies with letrozole have not been reported in other studies. Rate of multiple gestations with letrozole in other studies with 2.5 and 5 mg letrozole was zero (3, 4, 7, 9, 21).

This may be due to a larger sample size and simultaneous consumption of metformin with letrozole in the present study. Compared to other methods of ovulation induction the multiple pregnancy rate is approximately 10% with CC, and about 15-25% with gonadotropin. However, in this study the rate of multiple pregnancies with 2.5 mg letrozole was less than CC and with 5 mg letrozole it was less than gonadotropins.

As seen in Figure 1 and 2 ovulation and pregnancy increase with the combined use of metformin and incremental doses of letrozole, but the speed of this increase is slower in 7.5 mg dose of letrozole. 

This response to letrozole is similar to response to clomiphene. Most women who respond to clomiphene do so at either the 50mg (52%) or 100mg (22%) dose level. Higher doses can sometimes succeed when lower doses fail (11). This study showed with at least 6-8weeks of treatment with metformin alone and then addition of letrozole we can reach to a considerable pregnancy rate in CC-R PCOS patients.

Thus with regard to the good responses of CC-R patients to a combination of metformin with incremental doses of letrozole from 2.5 mg to 7.5 mg, lower rates of twin pregnancy than gonadotropins and no cases of ovarian hyperstimulation, we suggest that treatment should begin at first with lower dose of letrozole (2.5 mg) and could increase to higher doses (5 and 7.5 mg) depending on the patient response before considering more aggressive therapeutic alternatives such as gonadotropins. Thus metformin-letrozole is a valuable treatment when patients have financial constraints that preclude ovulation induction with HMG. 
